# Spectroscopic Techniques versus Pitot Tube for the Measurement of Flow Velocity in Narrow Ducts

**DOI:** 10.3390/s20247349

**Published:** 2020-12-21

**Authors:** Francesco D’Amato, Silvia Viciani, Alessio Montori, Marco Barucci, Carmen Morreale, Silvia Bertagna, Gabriele Migliavacca

**Affiliations:** 1 CNR-INO, Area CNR, Via Madonna del Piano 10, 50019 Sesto Fiorentino, Italy; francesco.damato@ino.cnr.it (F.D.); alessio.montori@ino.cnr.it (A.M.); marco.barucci@ino.cnr.it (M.B.); 2 Innovhub Stazioni Sperimentali per l’Industria srl, Via G. Galilei 1, 20097 San Donato Milanese, Italy; carmen.morreale@mi.camcom.it (C.M.); silvia.bertagna@mi.camcom.it (S.B.); gabriele.migliavacca@mi.camcom.it (G.M.)

**Keywords:** laser flow meter, Pitot tube, flow speed, time of flight, dilution method, flow simulation, flow turbulence, gas sensing applications

## Abstract

In order to assess the limits and applicability of Pitot tubes for the measurement of flow velocity in narrow ducts, e.g., biomass burning plants, an optical, dual function device was implemented. This sensor, based on spectroscopic techniques, targets a trace gas, injected inside the stack either in bursts, or continuously, so performing transit time or dilution measurements. A comparison of the two optical techniques with respect to Pitot readings was carried out in different flow conditions (speed, temperature, gas composition). The results of the two optical measurements are in agreement with each other and fit quite well the theoretical simulation of the flow field, while the results of the Pitot measurements show a remarkable dependence on position and inclination of the Pitot tube with respect to the duct axis. The implications for the metrology of small combustors’ emissions are outlined.

## 1. Introduction

Flow measurement in stacks is of fundamental importance in the assessment of pollutant emissions, because when combined with the measurement of the concentrations, it provides the mass flux of any emitted pollutant. Inside stacks flow is not laminar in most cases and the length of straight and unobstructed pipe sections, available for measurements, is often not enough to allow the full development of a regular velocity profile. Consequently, velocity and flow measurements are often affected by large uncertainty. Small ducts (inner diameter ≤ 50 cm) are more influenced than larger ones by perturbations, even due to the presence of sensors. Ducts can include curves, scrubbers, swirlers, extraction ports, probes and other devices, which can perturb the movement of the exhaust gases. Another issue is related to the presence of pollutants in the gas stream that can produce fouling or corrosion of the measuring devices. For example, particulate matter, often present in high concentration in many plant exhausts, such as biomass boilers, can obstruct the orifices for measuring the differential pressure or clog the moving parts of an anemometer. A recent review paper [1] addresses the origin of random and systematic errors for in-stack velocity measurements using Pitot tubes, how these problems are treated in the pertaining international standards and what effect they may have on the uncertainty of the measurements of pollutant emissions. From this survey, it emerges that, in the presence of cyclonic flows, the use of S-type Pitot tubes can result in errors of up to 12%, due to non-axial flows. A similar error can be produced by the misalignment of the Pitot tube during the measuring procedures. The errors associated with velocity measurements, using S-type Pitot tubes in cyclonic flows, have been investigated in detail also in [2] by means of computational fluid dynamic (CFD) modelling. Different pipe configurations, producing different flow patterns have been studied and the associated errors have been analyzed. In particular, the authors pointed out that, in presence of an asymmetric velocity profile, typically with swirling flows, the maximum velocity in a cross-section of stack describes a spiral along the duct. In this case, the results of the measurements depend on the location of the measuring point, whose best position is unpredictable. Another source of error, associated with the use of S-type Pitot tubes and examined in [2], is due to the inclined gas velocity vectors in presence of non-axial velocity components: the contribution of this error, estimated under the specific conditions simulated in [2], is up to 2.5%. Measurements were carried out in a wind tunnel, in different experimental conditions and geometries in [3], yielding similar results.

Several techniques are available for measuring in-stack velocity and flow rate: most of them are described in two international technical standards: EN ISO 16911-1:2013 [4] and EN ISO 16911-2:2013, the former focuses on manual and the latter on automatic methods. The most commonly used approaches make use of Pitot tubes or vane anemometers to determine the flow velocity either on a single fixed point or on a grid of points on a measuring plane. The other methods described in [4] include a tracer gas dilution technique and a calculation procedure, based on the energy consumption of the combustion plants. Both of them provide directly the gas flow rate and indirectly the average velocity in the stack. The tracer dilution technique follows a very basic approach, because it determines the flow rate using just two physical quantities: the mass flow rate of the injected tracer and the concentration of this tracer in the gas stream. The energy consumption approach relays on the possibility to measure the flow rate and composition of the fuel and the oxygen content in the fumes, continuously and very accurately. The Transit Time (TT) tracer gas method is another possible approach, briefly described in EN ISO 16911-1. This method allows determining the average velocity of the flowing gas within a portion of a duct having a constant cross-section. Many different solutions may be adopted to put this method into practice, which make use of a tracer gas which follows the gas flow and which can be measured with a sufficiently high time rate. For this technique to be adopted, some constraints should be fulfilled. It should be possible to inject a tracer inside the stack, as homogeneously (with respect to the transverse section of the duct) as possible. The tracer should not be naturally present in the atmosphere, should not interfere with the normal operation of the plant, should be neither poisonous or toxic, nor environmentally detrimental. It should not be a byproduct of the plant itself. For an accurate measurement the maximum response time of the detection technique must be ∼10÷100 ms, to be negligible compared to the rise time of the concentration of the tracer, which is in general ∼second. Short half-life radioactive tracers are often considered the most suitable, because they can be detected through the duct walls without the need for probes, ports or windows, but the use of these tracers is often restricted by national legislation. A detection technique that measures the average concentration of the tracer on a cross-section is to be preferred to a point measurement, because it reduces the effects of an improper mixing. Optical and laser techniques exist since long for the measurement of gas flows: Laser Two Focus [5], Doppler effect [6], Particle Imaging Velocimetry [7]. Laser Two Focus investigates very small regions (0.25 mm × 0.25 mm × 0.25 mm) at time, so requiring some time, and moving optics, to obtain a raster image. Laser Doppler Velocimetry provides information along a direction at an angle with the stack axis. In order to keep this angle as small as possible, inclined beams are required. In ref. [6] a setup with two measurement channels is shown. Particle Imaging Velocimetry requires the injection in the stack of particles with suitable dimensions, a pulsed, high power laser source, and a fast, high-resolution camera. Finally, a careful calibration procedure must be carried out. TT and Dilution techniques are much simpler, requiring low power laser sources and standard detectors, with straightforward analysis procedures. Spectroscopy is particularly suitable for the TT tracer gas method. In this case, the tracer should feature optical absorptions in wavelength regions where user-friendly laser sources are available, and these absorptions should be sufficiently strong to reduce the tracer concentration to very low levels (≤1‰).

In the framework of the EMPIR Project IMPRESS II, a laser-based device, presented in this article, has been developed, which allows us to perform both dilution and TT measurements of the in-stack velocity. The two methods have been tested and cross-validated in a stack simulator, having a small section, under strongly cyclonic flow conditions. The flow pattern inside the test rig has been simulated through CFD modeling, using Ansys^®^ Academic Fluent, Release 15.0, ANSYS, Inc., Canonsburg, PA, USA in order to get a better understanding of the experimental conditions. In parallel, fixed point measures, using an S-type Pitot tube, have been carried out and compared under conditions which are particularly critical for using a Pitot tube, in order to evaluate the possible bias between this conventional method and the other two optical techniques.

## 2. Optical Detection

In order to adopt any optical technique, we had to choose the tracer molecule. Following the constrains described above, we selected acetylene (C${}_{2}$H${}_{2}$). It has a strong absorption band around 1520 nm, which is one of the regions of optical telecommunications, and both laser sources and optical devices are available off-the-shelf. It is not toxic, and there is no risk of explosion at concentrations below 1‰(Low Explosion Level for acetylene is 2.5% [8]).

A possible drawback is that it can be produced during incomplete combustion of methane [9]. However, most of our measurements were carried out when heating the air inside the stack by means of electrically driven resistors, so the risk of interferences was avoided. In a real case, other fuels than methane could be used and, even in the case of incomplete combustion of methane, the injection of an extra amount of acetylene can be easily detected. In order to verify which concentration should be used in the measurements, to have a sufficient Signal-to-Noise Ratio, we examined the behavior of the absorptions in the temperature range of 285–385 K.

Molecular absorptions are described by the Beer-Lambert law:

(1) $$I_{out}=I_{in}\cdot e^{-\alpha L},$$

where $I_{out}$ and $I_{in}$ are the powers of the light beam after and before crossing the sample, respectively; *L* is the length of the sample, and

**Figure 1:**
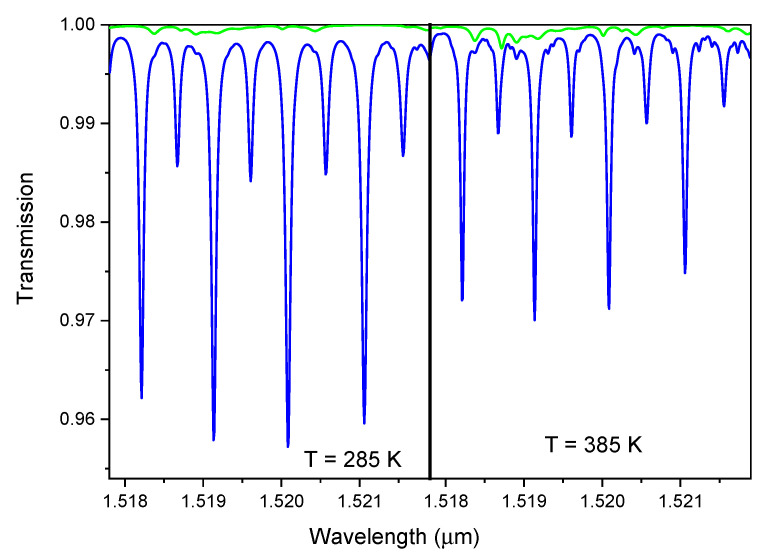
Comparison of the transmission spectra of an exhaust including H${}_{2}$O 10%, CO${}_{2}$ 10%, O${}_{2}$ 4% (green line), and the same mixture with addiction of C${}_{2}$H${}_{2}$ 1‰ (blue) at P = 1 Bar, L = 0.6 m, T = 285 K (**left**) and T = 385 K (**right**).

(2) α=S·g(ν)·n,

where *S* (cm/molecule) is the absorption strength, g(ν) (cm) the shape of the absorption (area normalized to 1), and *n* (molecule/cm${}^{3}$) the density of the absorbing species. One of the consequences of this behavior is that the absorbance of a transition depends on temperature, as both linestrength *S* and lineshape g(ν) are sensitive to temperature. Figure 1 shows the comparison of the transmissions, in a narrow wavelength range, at 285 and 385 K, at P = 1 Bar, L = 0.6 m, of a simulated exhaust mixture including H${}_{2}$O 10%, CO${}_{2}$ 10%, O${}_{2}$ 4% (green line), and the same mixture with addiction of C${}_{2}$H${}_{2}$ 1‰ (blue) [10].

It is evident that linestrengths decrease with increasing temperature. The best choice with respect to interference from other molecules is the line close to 1.521 μm, even if it is not the most intense one. Its strength decreases by about 38% at 385 K (with respect to 285 K), nevertheless, the green line in Figure 1 is very close to transmission 1 (i.e., unperturbed transmission) at both temperature conditions. Finally, a concentration around 1‰ is sufficient to produce absorptions of a few %, easily detectable by any optical technique. Actually, we easily worked at levels about one order of magnitude less than this. Due to all these features, and because suitable laser sources are commercially available at this wavelength, the transition at 1.521 μm should be selected in real operation conditions. As a matter of fact, in many of the present measurements we used hot air, or room temperature air, rather than exhaust gases, so the concentrations of water and carbon dioxide were the atmospheric values. For this reason in our tests we used either the absorption at 1.521 μm, or the more intense one at 1.520 μm.

As for the detection technique to be used, our choice was between Direct Absorption (DA) and Wavelength Modulation Spectroscopy (WMS) [11], depending on the kind of flow measurement. In DA the power of a light beam, transmitted across a sample, is converted by a detector into a voltage or current. When sweeping the laser wavelength, the absorption profile is retrieved. The analysis of this profile yields the absolute value of the concentration, provided that pressure, temperature, pathlength and molecular linestrength are known. In WMS, a modulation is applied to the laser wavelength at a period $\tau_{m}$ much lower than the sweep time $\tau_{s}$ (typically $\tau_{m}\leq 10^{-3}\cdot \tau_{s}$). The detector signal is demodulated at a multiple of the modulation frequency. The most used choice in commercial devices is a demodulation at twice the modulation frequency. In this case, the signal is approximately the second derivative of the absorption profile, and the central peak is proportional to the density of absorbing molecules. WMS exhibits a better signal-to-noise with respect to DA [12]. In a previous paper [13] we demonstrated the calibration problems of WMS when the composition of the sample varies, in particular because of large percentages of carbon dioxide and water, problems which do not occur with DA [14]. As a matter of fact, the two different optical flow measurements (TT and dilution) feature different calibration issues: TT is related to the time evolution of the concentration of the injected tracer and not to the absolute value of the mixing ratio. On the contrary, dilution requires an accurate measurement of the tracer mixing ratio. For these reasons we chose DA for dilution, and WMS for TT measurements.

## 3. Materials and Methods

### 3.1. Layout of the Test Rig

**Figure 2:**
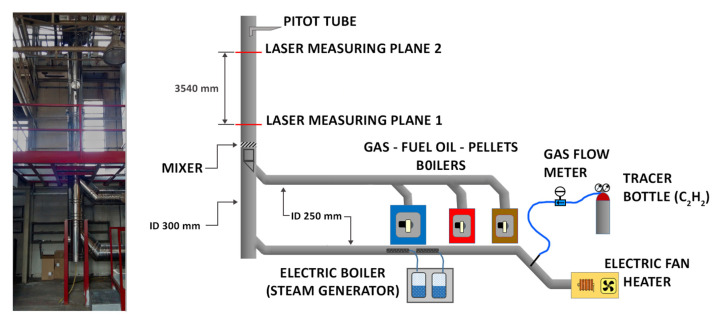
General layout of the stack simulation facility used in this study.

The measurements were carried out at the Innovhub premises in San Donato Milanese (MI), Italy. The testing facility, used to validate the proposed technique, is a small-scale stack simulator, consisting of a flue generator and a vertical duct, where two measuring planes are available. A detailed description of the plant is shown in Figure 2. Real exhaust gases can be produced using one of the boilers connected to the plant; in particular, a 300 kW gas boiler, a 150 kW fuel oil boiler and a 100 kW biomass boiler are available. Otherwise, simulated exhausts can be produced using a fan, an electric heating unit and a steam generator; the flue gases produced this way can be more easily modulated since each of the parameters (namely flow rate, temperature and moisture content) can be set within a relatively large range. On the contrary, when a boiler is used, the composition, temperature and flow rate of the exhausts are mostly fixed, depending of the power and feed of the boiler, just minor adjustments can be produced by acting on the boiler’s settings. Both the real exhausts and the simulated fumes are conveyed through horizontal ducts to the same vertical stack, having a constant circular section of 300 mm of internal diameter (ID) and being 12 m high totally. The stack is a double wall stainless steel duct with a 25 mm thick thermal insulation. The gas is conveyed into the stack through a 45${}^{\circ }$ inclined insertion duct; a coaxial section, consisting of a 250 mm inner duct, allows straightening the velocity field. Downstream, a screw-shaped mixing section is placed, in order to force the stream coming from the boilers to mix with the one coming from the fan, when both are necessary at the same time. The first measuring plane is located about 3 diameters downstream of this section. The second one is placed 3.54 m higher (i.e., 12 ID downstream). Both measurement points are equipped with two opposite flanged ports, housing the optical windows. About 5 m higher the fumes are released in the atmosphere. Additional ports for inserting probes used for the analysis of the gas composition and for the measurement of point velocity are located close to the two main measuring planes. The Pitot tubes used in the present work were placed 0.3 m above the upper measuring point. The injection point of the tracer was placed as far as possible from the measuring planes, so that the better possible mixing could be obtained. A small perforated pipe, inserted into the main duct close to the flue generator, was used to distribute the tracer in the mainstream. A gas bottle, containing the pure tracer, was connected to the injection point through a flexible PTFE tube; a pressure regulator was used to set the pressure of the tracer at the injection point. A quick connection hose was used to manually connect/disconnect the tracer carrying tube with the injection pipe at the beginning of each TT test. On the other hand, a calibrated mass-flow meter was used to feed a known amount of tracer during the dilution tests. The tracer is mixed with the fumes in the straight horizontal duct (about 10 m long), a further and more intense mixing is produced in the mixing section of the stack, previously described.

### 3.2. Optical Platform

The optical platform, based on the first version described in [11], was developed in order to adapt it to provide both DA and WMS signals for two measurement points inside the stack and for a reference arm.

#### 3.2.1. Optics

**Figure 3:**
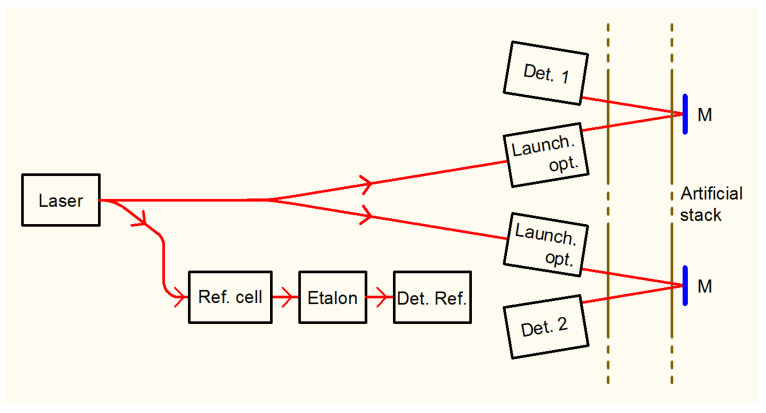
Block diagram of the optics. M: flat Mirror. The angle between the two couples of beams inside the stack is exaggerated for the sake of clarity.

**Figure 4:**
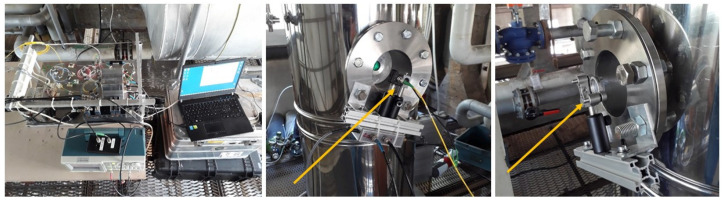
(**left**): main plate of the mechanics, together with the waveform generator and the laptop; (**center**): launching optics, driven by the yellow optic fiber, and detector; (**right**): steering mirror, which reflects the laser beam onto the detector.

The optical scheme is described in Figure 3. The laser source is a DFB fiber laser with an emission wavelength at 1521 nm (Eblana Photonics EP1521-0-DM-B01-FA, butterfly package, pigtail output). Immediately out of the laser, a beam splitter (Thorlabs TW1550R1A2) takes 1% of the beam to a fiber-coupled reference cell (Wavelength References C2H2-12-H(5.5)-50-FCAPC, 50 Torr, 5.5 cm optical path) and home-made BK7 etalon, length 30.75 mm, Free Spectral Range 3.25 GHz, to obtain a relative frequency scale. The remaining 99% is 50:50 split (Thorlabs TW1550R5A2) into two beams, which are sent to the measurement points. As shown in Figure 4 Center, the launcher is very close (2 cm) to the detector. The beam crosses the stack and is reflected by a plane mirror, directly onto the detector. As the stack diameter is 30 cm, and inside the stack the distance between the two beams is less than 2 cm, we can affirm with a good approximation that we are probing the tracer concentration along a diameter of the stack. It’s worth noting that both optical measurement channels are necessary for the TT measurement only, as dilution is measured in steady-state conditions, and one channel only could be used. Yet, once the experimental apparatus is completely installed, the presence of two optical lines makes it possible to have a redundancy in the dilution measurements.

#### 3.2.2. Mechanics

Mechanics is divided into three parts. The first is an aluminum breadboard that hosts the electronics, the laser and the reference optics (Figure 4 Left). The breadboard is framed into a structure that fits a plastic suitcase for easy transportation. The other two parts of the mechanics are mounted onto the stack. The launching/receiving optics can be seen in Figure 4 Center. The laser beam is shot across the stack, onto a mirror (Figure 4 Right), which directly reflects the beam onto the detector, for a total of two passes inside the stack. The mechanical stability of the system relies on the flanges protruding out the stack. All the optical ports are sealed by 1” anti-reflection coated BK7 windows (Thorlabs WG11050-C).

#### 3.2.3. Electronics

**Figure 5:**
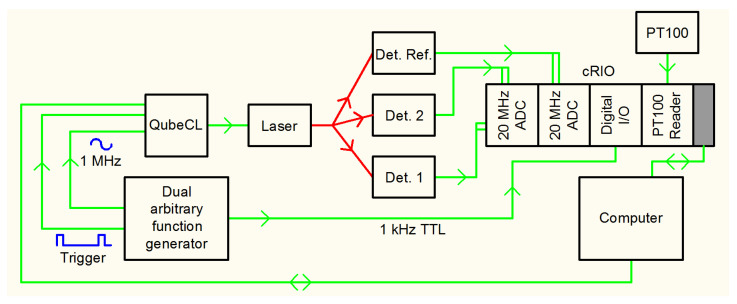
Block diagram of the electronics.

A block diagram of the electronics is shown in Figure 5. The laser is current-supplied and temperature stabilized by a commercial driver (ppqSense QubeCL). A National Instruments crate (cRIO 9067) hosts: two 4-channels, 20 MSamples/s acquisition rate each, 14 bits vertical resolution ADC plug-in’s (NI-9775); a programmable, 4-channels digital I/O plug-in (NI-9402); and a 4-channels PT100 reader (NI-9217) for housekeeping. An important feature of this crate is its FPGA, Xilinx Zynq-7000, with 85.000 logic cells and 106.400 flip-flops. A Tektronix double output Arbitrary Function Generator 3022 provided: the trigger (1 kHz) for the QubeCL to start the sawtooth ramp to sweep the laser frequency across an absorption profile, the TTL output signal for the digital I/O plug-in which starts the acquisition on the rising edge of the TTL, and the high frequency (1 MHz) sinusoid used for WMS. The latter signal was converted by the QubeCL into current, and added, together with the ramp, to the bias current of the laser. We used three detectors, Hamamatsu InGaAs mod. G12180-210A, 1 mm diameter, 2-stage Peltier cooling, 40 MHz cutoff frequency, two for the detection points and the third for the reference arm. Each detector is equipped with two outputs, low pass (<100 kHz) and high pass (>500 kHz) filtered. The outputs of the detectors are acquired by six channels of the NI-9775 modules. The low pass signals are used for DA and the high pass signals are used for WMS.

#### 3.2.4. Software

Once set the function generator and the laser driver, the procedure of synchronization, acquisition and storage is carried out by the FPGA of the NI cRIO. We wrote a dedicated LabVIEW program for data acquisition and for the implementation of a digital lock-in amplifier, according to well-known principles and routines [15,16,17]. The lock-in can operate in single and dual-phase mode. The modulated signals are acquired by the 20 MS/s ADCs for a time corresponding to an integer multiple *N* of the modulation period. The higher *N*, the higher the integration time. The actual value of *N* is chosen taking into account the rise/fall time of the concentration peak. The deconvolution procedure is carried out by the FPGA: the signals are multiplied by a reference sinusoid and then integrated during the N periods. In single-phase mode, the phase of the reference signal can be adjusted, in order to maximize the output. In dual phase-mode, a quadrature detection can be performed, multiplying the acquired signals by two reference signals, 90${}^{\circ }$ out of phase with respect to each other, and then taking the module and the sign.

The software allows all these choices, and stores data in the USB memory stick connected to the cRIO.

### 3.3. Optical Methods

#### 3.3.1. Direct Absorption and Dilution Method

The dilution measurements consist of a repeated laser wavelength scan (without the sinusoidal modulation) across the selected C${}_{2}$H${}_{2}$ absorption line. Each scan is acquired as a set of 20,000 points for each channel, in 1 ms. A binning over 100 points reduces the points per ramp to 200. Binning, and average of the scans, are performed by the FPGA, producing a spectrum every 28 ms. These spectra are saved in the computer. The final waveform is fitted by using the theoretical Voigt absorption profile. According to the procedure described in [18], the mixing ratio can be inferred by the fitting parameters, provided that temperature, pressure, molecular linestrength and path-length are known. Pressure and temperature are measured by sensors included in the Pitot probe (accuracy 0.05% and 0.3%, respectively), the linestrength is reported in HITRAN molecular database [10] with accuracy 1% and the pathlength was measured to be 580 ± 1.5 mm.

**Figure 6:**
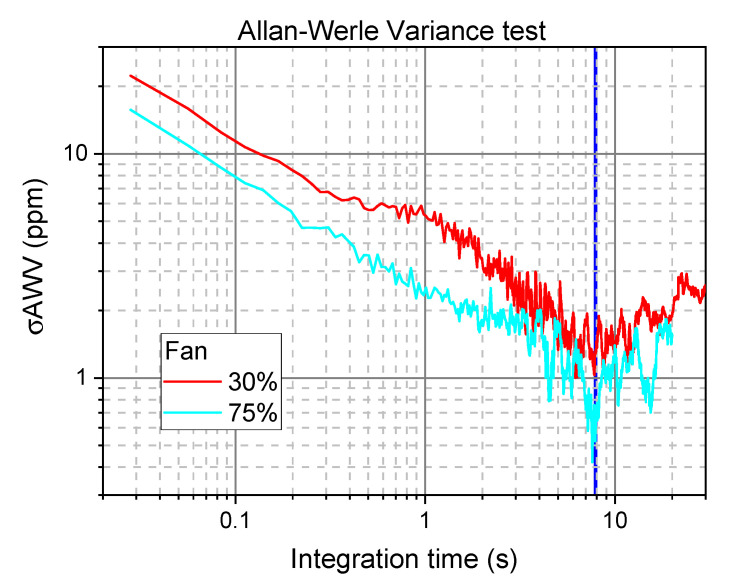
Allan-Werle test of the system at two different fan speeds, with respect to maximum.

**Figure 7:**
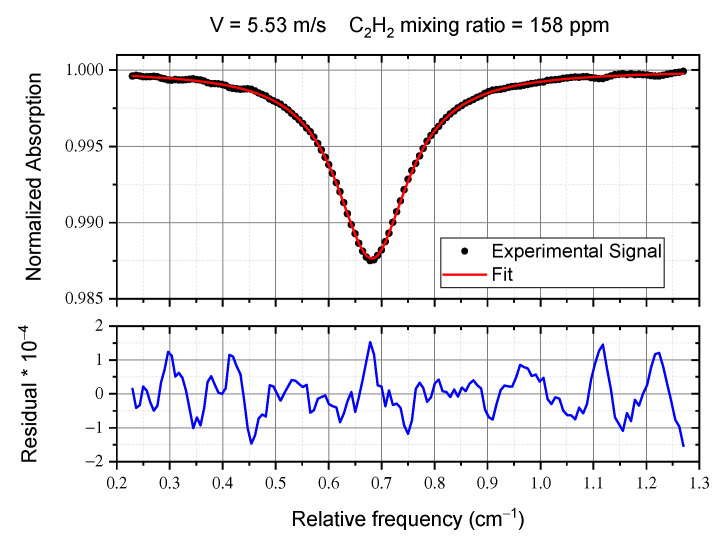
Plot of a direct absorption acquisition and its analysis.

In order to verify the quality of our data, and to decide the best integration time for our measurements, we performed the Allan-Werle Variance [19,20] on mixing ratio measurements. Figure 6 shows the results obtained for two different speeds of the fan (expressed as % of the maximum speed). The best integration time results to be 8 s, corresponding to an Allan-Werle Variance $\sigma_{AWV}$ of 1.5 ppm at 30% and 0.9 at 75%. The slight difference can be due to the different measurement conditions. It is worth noting that this is the Allan-Werle test of the whole system, formed by the fan, the mass-flow meter and our optical apparatus. Dilution measurements are stationary, so, according to the Allan–Werle analysis, we can further average over 285 scans, for 8 s total measurement time. Figure 7 shows the averaged absorption profile, with the fit curve and the residual.

The calculation of the flow speed is performed by measuring the steady-state concentration of the tracer, when the amount of injected tracer per unit time and the transverse section of the duct are known, according to the formula:

(3) $$V=\frac{T}{CS}$$

where *V* is the velocity (m/s), *T* is the flow (m${}^{3}$/s) of the injected tracer, *C* is the mixing ratio measured downstream, and *S* (m${}^{2}$) is the transverse area of the duct.

#### 3.3.2. Wavelength Modulation Spectroscopy and TT Method

**Figure 8:**
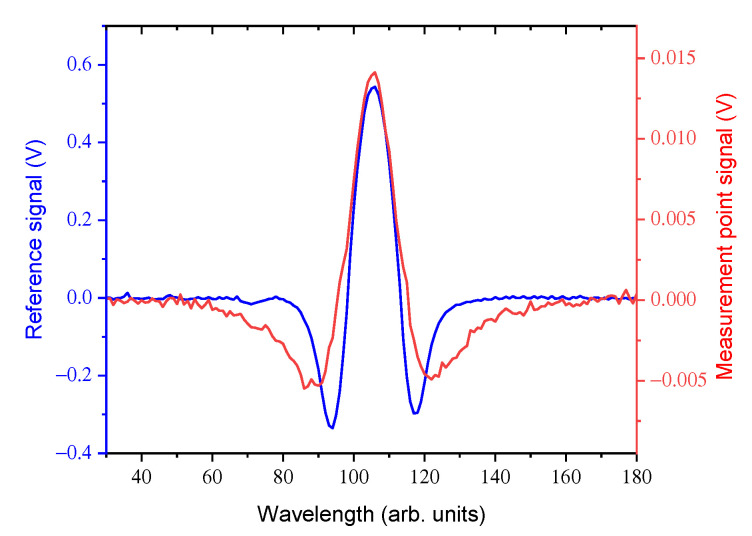
Wavelength Modulation Spectroscopy (WMS) signals from the reference cell (blue) and one of the measurement points along the stack (red).

To perform Second Derivative WMS, the fast modulation (1 MHz) is added to the laser sweep (1 kHz) and the acquired signal is deconvolved at 2 MHz. An example of WMS signals from the reference cell and from one measuring point along the stack is shown in Figure 8, where the different shapes of the two waveforms are due to the different pressures inside the cell and the stack. The peak values of the profiles in Figure 8 are proportional to the concentration. In the TT method, the time profile of the peak heights is acquired at two different measuring points (reference is only used to check that the laser wavelength is not drifting), when a concentration burst of the tracer is inserted inside the stack. In order to have the maximum acquisition rate, the laser wavelength sweep is stopped and the laser is set at the peak of the absorption. In principle, it would be possible to implement an active stabilization routine, based on an odd derivative (demodulation at an odd multiple of the modulation frequency). As a matter of fact, due to the stability of the laser driver, active stabilization was not necessary, and emission stability was maintained for a time larger than the 10 s of the measurement duration. With this procedure, the acquisition rate is 1 kHz, sufficient to follow the time profile of C${}_{2}$H${}_{2}$ inside the stack.

**Figure 9:**
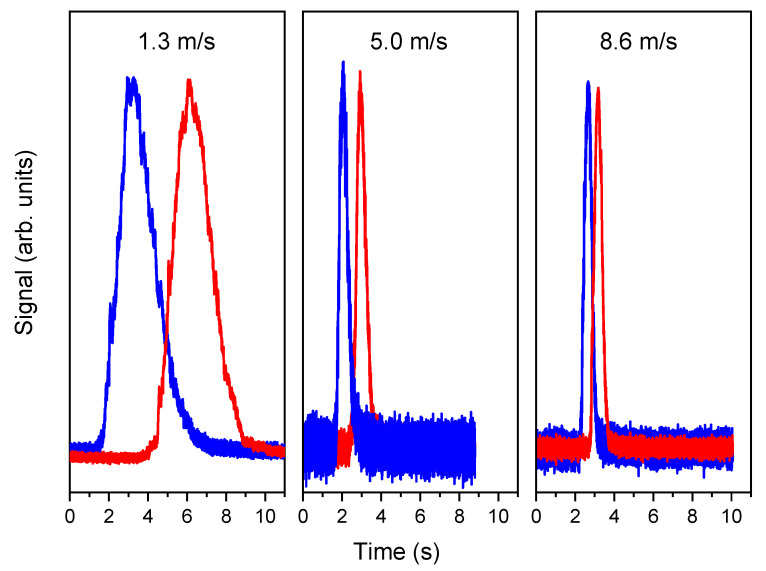
Set of Transit Time (TT) measurements at three different flow speeds, at the lower (blue) and upper (red) measurement points.

To obtain the concentration burst, the tracer gas line was closed and opened with a delay as short as possible. An example of the TT measurements, at two different points of the stack, at three different flow speeds, is reported in Figure 9. The velocity can be obtained by calculating the time delay between two corresponding timestamps of the red and blue curves of Figure 9. The selection of the timestamps is not a minor issue and it will be analyzed in the next section. The geometrical distance between the two measuring planes (3.54 m) divided by the delay yields the average gas velocity.

### 3.4. CFD Modeling

CFD calculations were used to simulate the flow field inside the stack, comparing the model with the experimental results, and driving further measurements. The commercial software Ansys^®^ Academic Fluent, Release 15.0 has been used for this calculation. It solves conservation equations for mass and momentum, according to an Eulerian approach, while an additional equation for energy conservation is solved to account for heat transfer processes. For flows involving species mixing or reactions, a species conservation equation is solved for each one and additional transport equations are also solved when the flow is turbulent.

In this model, the turbulent viscosity $\mu_{t}$, assumed as isotropic, is computed by combining the turbulent kinetic energy *k* and its dissipation rate ϵ as follows:

(4) $$\mu_{t}=\rho C_{\mu }\frac{k^{2}}{\epsilon },$$

where ρ is the density and $C_{\mu }$ is a constant.

A tetrahedral unstructured grid, consisting of 2.6 M cells, has been set up; the standard kϵ model of turbulence has been adopted; the time-dependent simulations have been carried out using a fixed time step of 0.05 s. The composition, velocity and temperature of the gas stream have been set at the inlet, as boundary conditions for the numerical solver of the model equations. A homogeneous distribution of the tracer has been assumed on the inlet section at time zero conditions. Then, the average concentrations of the tracer on two straight lines corresponding, in the model grid, to the laser beam path lines in the real plant, have been computed for each time step, by averaging the tracer concentration calculated in each cell laying on these lines. These average concentrations are directly comparable with the detected signals.

#### 3.4.1. Simulation of the Tracer Concentration Profile

**Figure 10:**
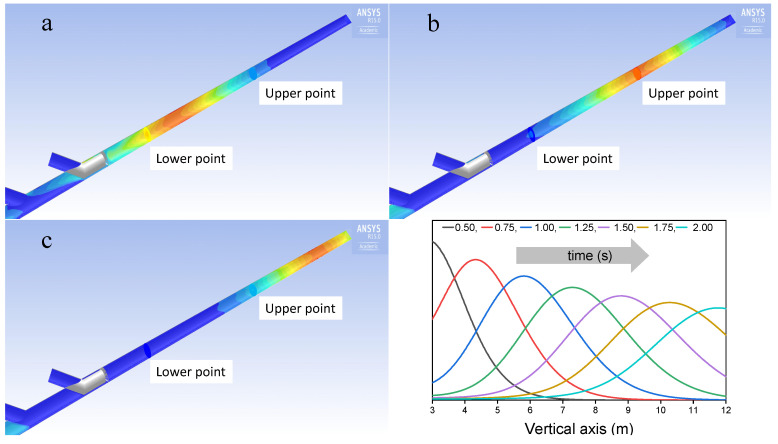
Computational fluid dynamic (CFD) tracer concentration profile along the stack axis (average velocity 6 m/s, T = 105 ${}^{\circ }$C). Time origin is the transit time of the tracer at the upper junction (grey in the figures). (**a**) 0.75 s; (**b**) 1.25 s; (**c**) 1.75 s.

By using CFD simulation, the time profiles of the concentration of the tracer were calculated at any point along the stack, in order to make a comparison with experimental data and to select the best method to calculate the gas velocity during TT measurements. The simulated concentration profile varies along the vertical axis, as a consequence of the progressive mixing of the tracer. This makes the curves, at the two measuring planes, not perfectly overlapping and a specific criterion has to be defined in order to select the two reference timestamps (one for each curve) to be compared to measure the time delay for velocity calculation. Different choices, for these points, produce slightly different outcomes in terms of velocities, which means that this choice is an essential aspect of the method. The expected profiles of the concentration of the tracer, along the vertical axis of the stack, as calculated through CFD simulations, are reported in Figure 10, for an average gas velocity of 6 m/s and a temperature of 105 ${}^{\circ }$C, as well as the corresponding bi-dimensional distributions, on a vertical plane, at 0.75, 1.25 and 1.75 s, as explained in the caption.

**Figure 11:**
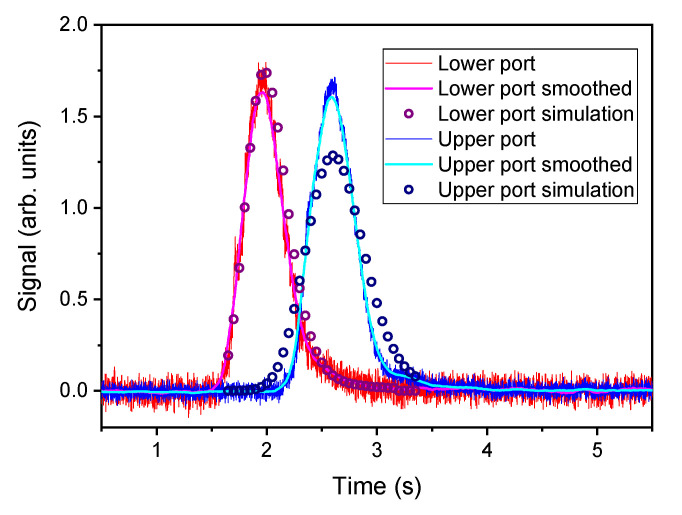
Comparison between simulation and experimental data for concentration profiles at the average velocity 6 m/s, T = 105 ${}^{\circ }$C).

It can be observed that the increase/decay curve spans for a long distance along the vertical axis; from this point of view, these conditions are quite different from an ideal plug flow. The shape of the curve actually changes along the axis: its slope decreases and the peak broadens, as expected. In Figure 11 experimental and model results are compared: the signals of TT measurements collected at the lower and upper measuring planes are plotted together with the results of CFD simulation (circles) at the same test conditions. The broadening effect is produced by the combination of the bulk velocity of the flow and the diffusion velocity of the tracer molecules. The second peak in the simulation shows a larger broadening effect compared to the experimental results. It can be assumed that the adopted model of turbulence overestimated the turbulent diffusivity of the system. More advanced modeling, for instance including Large Eddy Simulation (LES) could have produced a better matching, but a fully detailed simulation is outside the scope of this work.

**Figure 12:**
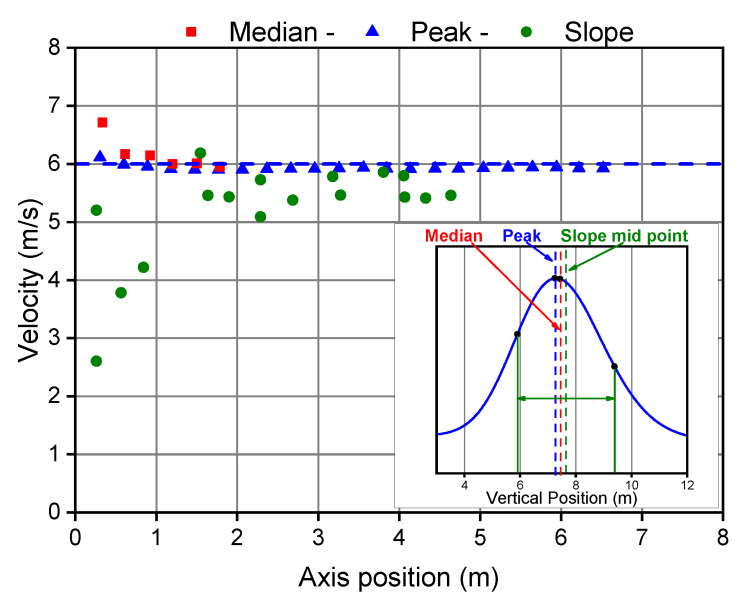
Calculated velocities using different points on the simulated tracer concentration curves at different times (average velocity 6 m/s, T = 105 ${}^{\circ }$C).

Molecular and turbulent diffusion produces an apparent velocity, which gives incorrect results if the reference timestamps of each curve are not selected properly. As far as an isotropic diffusivity is assumed, each molecule diffuses randomly in any direction: molecules mainly diffusing in the flow direction move faster than average fluid-dynamic velocity, while molecules diffusing mainly against the flow direction move slower than average fluid-dynamic velocity. Consequently, these points are not suitable for measuring the bulk velocity of the flow. The centroid of the tracer spike should be identified in order to get a correct result from the TT technique; EN ISO 16911-1 states that the best choice is the median of the concentration distribution. A specific analysis has been carried out to compare different possible criteria. The simulated concentration distributions can be used to compare the results one can expect by applying different approaches of calculation on the same data. In Figure 12 the average gas velocity was estimated from three different sets of points, on the simulated curves: the peak of the curve, the median, and the mid-point between the maximum slope at both sides of the peak (the inset shows the location of these points). These calculations have been repeated for several axial distances, in order to evaluate the effect of the separation between the two measuring points. The best matching with the known average actual velocity is produced using the peaks. The velocities calculated based on the median are equally in good agreement with the expected result, except for very short distances when velocities would be overestimated. The sloping approach produces more erratic results, probably due to the fluctuations produced by the numerical derivation of the curves. It is noteworthy, anyway, that the three points, selected on each curve, are quite close to each other, because of the symmetry of these curves, while many different results may be expected in more asymmetric conditions.

#### 3.4.2. Simulation of Velocity Field Inside the Stack

**Figure 13:**
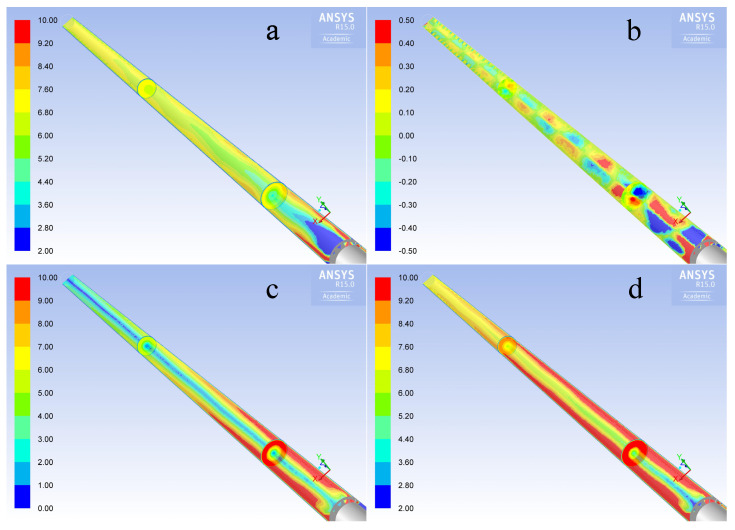
Simulated velocity field inside the stack simulator in all its components: (**a**) total, (**b**) axial, (**c**) radial and (**d**) tangential velocity. Units are m/s.

**Figure 14:**
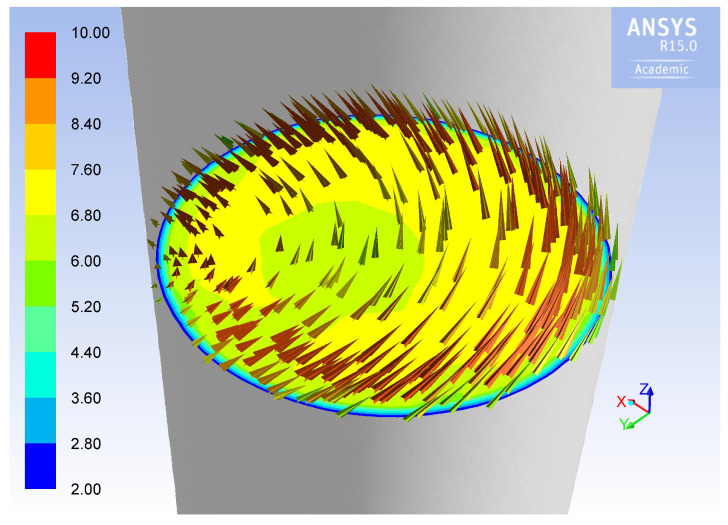
Simulation of the flow field along a horizontal section of the duct, corresponding to the Pitot port position. Units are m/s.

A simulation of the velocity field inside the stack was carried out in order to understand how critical is the positioning of the Pitot tube, due to the fact that the flow field inside the stack does not feature a cylindrical symmetry, but a complex behavior, as reported in literature [3,21]. Figure 13 shows the calculated components of the gas velocity along the stack. The velocity vectors on the horizontal section where the Pitot probe was installed are shown in detail in Figure 14: the flow field is strongly asymmetric and the vectors are inclined in a quite complex configuration. As a consequence, the location of the Pitot probe can critically affect the velocity measurements and deserves further investigation.

## 4. Measurements and Results

The experimental tests were carried out under different conditions of gas velocity, temperature and composition, in order to check the response of the three techniques and the effect of each parameter.

### 4.1. Pitot Probe Test

**Figure 15:**
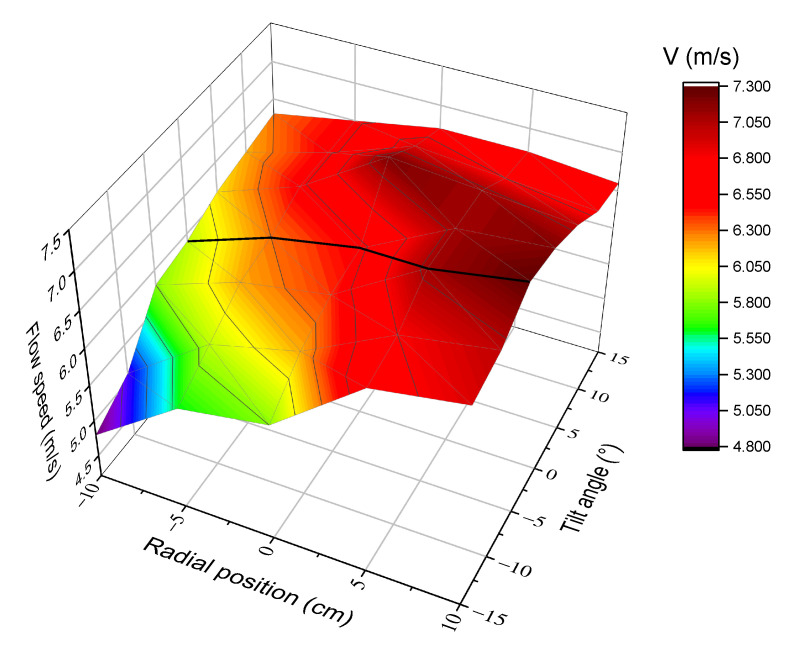
Plot of the flow field, along a diameter of the stack, with different Pitot angles with respect to the duct axis. Units are m/s.

We do not describe the working principle of Pitot tubes, it is enough to say that an S-type Pitot, connected to a micromanometric device, specifically designed for in-stack velocity and flow rate measurements was used in the first series of intercomparison tests. This device is designed to be integral with the probe and has an integrated inclinometer. The Pitot tube was placed 0.3 m downstream the upper measuring plane and velocity measurements were carried at a fixed point on the central axis of the stack, in accordance with EN 15259 for ducts having an internal diameter smaller than 0.35 m. Despite this, it is not possible to be sure that this is the best choice in the specific case of a cyclonic and asymmetrical flow, such as the one studied in the previous section. Hence, we decided to investigate this feature in deeper detail. We performed a series of measurements, using the Pitot tube only, testing a flow of air at room conditions. The Pitot probe was inserted inside the stack at different distances from the duct axis (negative values mean between the port and the duct axis, positive values mean beyond the duct axis), and at different angles with respect to the vertical direction. The results of this test are shown in Figure 15. It is evident that the velocities are not constant along a diameter. Moreover, when tilting the Pitot probe, even by a few degrees, the readings change. From this investigation, we concluded that the reading at the central position is approximately equal to the average velocity on that line and only if the probe is strictly vertical. Just a little displacement along the axis or a small rotation is enough to read a quite different velocity.

### 4.2. First Set of Measurements

The first set of measurements was carried out with ambient air at room temperature, in order to have a set of intercomparison data: for each flow fan-speed we recorded the velocity readings obtained by the Pitot, and the results of the dilution and the TT techniques. As dilution and TT measurements cannot be carried out at the same time, we used the fan speed (% with respect to maximum speed) as a reference for different series of measurements.

**Figure 16:**
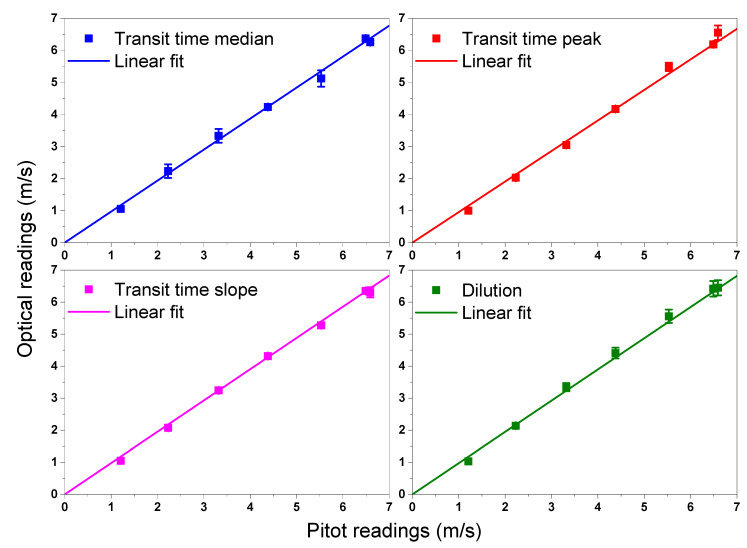
First set of flow velocity measurements, comparing Pitot tube with three different analysis of the TT signals and with dilution. Points are related to different fan velocities.

In order to perform dilution measurements, the injection was constant, measured by using a thermal-mass-flowmeter (Bronkhorst High-Tech model EL-FLOW, accuracy 3.5%), at the level of 3.7 ÷ 3.9 L/min ($6.17\times 10^{-5}÷6.50\times 10^{-5}$ m${}^{3}/$s). The concentration readings were in the range 136 ÷ 845 ppm, so well below 1‰, as explained in Section 2. In order to measure the time delay to calculate the velocity, we took the time stamp of the transit of a peak in different ways, described in Section 3.4.1: the median of the distribution of the signal, the peak and the mid-point of the maximum peak slopes. Figure 16 shows the readings of dilution and different time stamps of TT, vs. Pitot readings, for different fan speeds. For TT method each point is the mean value of a set of measurements and the error bars are the corresponding standard deviations. For the dilution method, each point is calculated according to (3), and the error bars are the accuracy of each measurement.

There is a very good correlation between the Pitot tube and the two optical techniques, whatever the analysis of the TT signals. In each graph of Figure 16 the first point is not taken into account for the linear fit. The reason is that the Pitot tube shouldn’t be used around 1 m/s, as its linearity is poor in this range. In fact, the average flow speed at the first point of each graph, read with optical techniques is 1.04 m/s, while the Pitot reading is 1.21 m/s, which is the largest discrepancy among Pitot and optical readings. In Table 1 the different slopes of the fit curves (i.e., the proportion coefficient with respect to Pitot tube) are reported. We can note that all the slopes are equal, within their uncertainties, and slightly less than unity. As the dilution technique is not affected by any fluid-dynamic effect, its readings can be assumed as the reference values and the very small difference with the TT results proves the reliability of the TT technique, and its independence of the selection of the line of sight.

### 4.3. Second Set of Measurements

**Figure 17:**
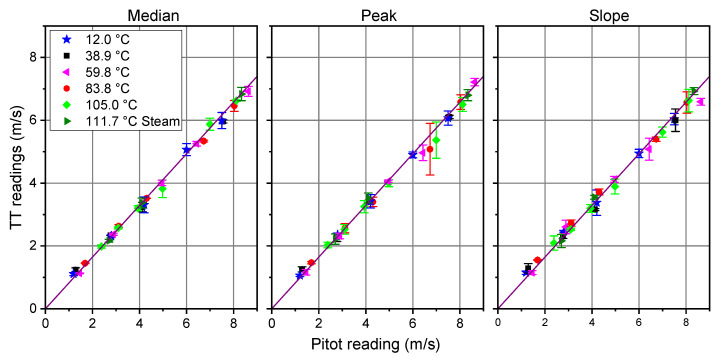
TT readings, with different time stamps, vs. Pitot readings, with manual setting of the Pitot probe for different temperatures and fan speeds. Steam flow rate at 111.7 ${}^{\circ }$C: 60 kg/h @ 2.7 m/s; 31 kg/h @ 4.1 m/s; 18 kg/h @ 8.3 m/s.

We carried out a second run of measurements, in order to investigate a wider range of experimental conditions, and the use of a conventional Pitot probe, adopted for in-field isokinetic sampling, manually aligned, both vertically and with respect to the duct axis, as routinely performed in real-life periodic measurements. This time the fluid was room air, heated using electric resistors. At the highest temperature, steam was added to room air, at a rate depending on the flow speed. For each temperature, the fan speed was set at different levels. In this set of measurements, the Pitot readings were compared with TT only. The results show very good linearity of the TT readings, at any temperature and gas composition, with respect to Pitot data, whatever the criterion for the time stamp of each peak (Figure 17). On the other side, it is evident that there is a systematic deviation between the TT readings and the Pitot results, whose ratio was measured to be 0.822 ± 0.002. This result is not surprising for a manually aligned probe, according to previous literature, and following our simulations and experimental tests. The amount of the discrepancy is in this case above 20%, which means that the most unfavorable conditions of the positioning of the Pitot probe were encountered here.

## 5. Discussion and Perspectives

We have applied two spectroscopic detection techniques to the measurement of flow in narrow ducts, and to the calibration of standard sensors and methods. Our multipurpose device was deployed in a stack simulator, proving to fulfill all the requirements for the above task. It is already stated in EN ISO 16911-1 that the dilution method is a valuable reference method, which is intrinsically free from wall effects, or from any perturbation due to the geometry of the duct, or to any objects inserted in the duct, inducing turbulence. On the contrary, the more turbulent the flow, the more homogeneous the distribution of the tracer. TT method, in particular the one implemented in this work, based on optical techniques, compares very closely with dilution.

The conventional procedure adopted for calibrating Pitot tubes is carried out ex-situ in a standardized wind tunnel using a primary reference device. It generally provides either a single calibration factor or a set of calibration factors for the different velocity ranges, used to calculate the point velocity from the readings of the differential pressure at the Pitot ends. Calibrated Pitot tubes are then used both to measure in stack velocity directly and to periodically calibrate other automated measuring systems, installed on the stacks to measure the flow rate on line. This study shows that this approach can produce inaccurate results when cyclonic flows in small ducts are involved, in particular:

1. the conditions of 5 internal diameters of straight duct upstream and 3 downstream of the measuring section, without elements disturbing the flow, may not be a sufficient guarantee to provide a regular velocity profile at the measuring plain;
2. single-point measurements may be non-representative of the average velocity, a multipoint technique should be required even for ducts having an internal diameter smaller than 0.35 m;
3. manual alignment of a Pitot tube is not accurate and stable enough, large deviations of the measured values may be produced by very small variations in the placement of the probe;
4. techniques relying on the bulk properties of the flow, such as the concentration of a tracer injected in the gas stream, are largely insensitive to the flow pattern and the local disturbances, producing linear and accurate results.

As a consequence of these considerations, the pertaining international standards, namely UNI EN 15259:2008, EN ISO 16911-1:2013 and EN ISO 16911-2:2013, should include specific warnings for small ducts, suggesting the use of reliable and robust techniques for the in-site calibration of the automated measurement systems, such as dilution based and transit time methods. Moreover, apart from periodical checks, the necessity to repeat the calibration every time a modification occurs in the duct, upstream the sensor should be introduced.

Table 2 compares the requirements of the different techniques used in this work. Pitot tubes are undoubtedly the simplest technique for flow velocity measurements, as they require an insertion port only, and no consumables. TT requires four optical ports. Dilution can be implemented either across the stack, or after gas extraction. In the first case, two optical ports are necessary, in the second case an extraction port (downstream the Pitot, to avoid any interference) and a heated line must be used. In both the latter cases, a tracer is required, which means consumables and an injection point. Despite the higher complexity, we proved that in narrow ducts it is necessary to add ports, or injection/extraction points, suitable for the application of more complicated techniques, for intercomparison and calibration. As a final remark, the described optical techniques are not so much time consuming, as they require one workday for set-up, measurement and packing.

## Figures and Tables

**Table 1:** Table of the proportionality coefficients between optical techniques and Pitot tube for different analysis procedures.

Technique	Ratio	Uncertainty
TT median	0.968	0.007
TT peak	0.97	0.01
TT slope	0.976	0.005
Dilution	0.97	0.01

**Table 2:** Comparison of Pitot tube, TT and dilution methods.

	Affected by Position	On-Site Calibration	Traces Gas	Optical Ports	Extraction
Pitot tube	YES	Compulsory	NO	NO	NO
Dilution	NO	NO	YES	YES/NO	NO/YES
TT	NO	NO	YES	YES	NO
